# Prompt engineering for accurate statistical reasoning with large language models in medical research

**DOI:** 10.3389/frai.2025.1658316

**Published:** 2025-10-13

**Authors:** Sifiso Vilakati

**Affiliations:** Department of Biostatistics, University of the Free State, Bloemfontein, South Africa

**Keywords:** prompt engineering, large language models, statistical reasoning, medical research, AI-assisted data analysis, evaluation frameworks, LLM-as-a-judge, statistical assumption checking

## Abstract

**Background:**

The integration of generative artificial intelligence (AI), particularly large language models (LLMs), into medical statistics offers transformative potential. However, it also introduces risks of erroneous responses, especially in tasks requiring statistical rigor.

**Objective:**

To evaluate the effectiveness of various prompt engineering strategies in guiding LLMs toward accurate and interpretable statistical reasoning in biomedical research.

**Methods:**

Four prompting strategies: zero-shot, explicit instruction, chain-of-thought, and hybrid were assessed using artificial datasets involving descriptive and inferential statistical tasks. Outputs from GPT-4.1 and Claude 3.7 Sonnet were evaluated using Microsoft Copilot as an LLM-as-a-judge, with human oversight.

**Results:**

Zero-shot prompting was sufficient for basic descriptive tasks but failed in inferential contexts due to lack of assumption checking. Hybrid prompting, which combines explicit instructions, reasoning scaffolds, and format constraints, consistently produced the most accurate and interpretable results. Evaluation scores across four criteria–assumption checking, test selection, output completeness, and interpretive quality confirmed the superiority of structured prompts.

**Conclusion:**

Prompt design is a critical determinant of output quality in AI-assisted statistical analysis. Hybrid prompting strategies should be adopted as best practice in medical research to ensure methodological rigor and reproducibility. Additional testing with newer models, including Claude 4 Sonnet, Claude 4 Opus, o3 mini, and o4 mini, confirmed the consistency of results, supporting the generalizability of findings across both Anthropic and OpenAI model families. This study highlights prompt engineering as a core competency in AI-assisted medical research and calls for the development of standardized prompt templates, evaluation rubrics, and further studies across diverse statistical domains to support robust and reproducible scientific inquiry.

## 1 Introduction

The rapid evolution of generative artificial intelligence (AI) has ushered in a new era for scientific research and knowledge production. From the early days of rule-based natural language processing to the advent of large language models (LLMs) such as OpenAI's GPT series, generative AI has demonstrated remarkable capabilities in understanding, generating, and analyzing human language. These models, trained on vast amount of data, can now summarize complex literature, and even generate code for statistical analysis, tasks that once required years of specialized training (He et al., 2025; Akhtar, 2024).

The history of generative AI is marked by exponential progress. Early systems relied on hand-crafted rules and limited datasets, producing outputs that were often rigid and contextually shallow (Schmidhuber, 2015; Norvig and Stuart, 2021). The introduction of neural networks and, later, transformer architectures revolutionized the field, enabling models to capture nuanced relationships in language and context (Munro, 1984). Today's LLMs, with billions of parameters and access to diverse knowledge domains, are increasingly being integrated into the workflows of medical researchers, clinicians, and statisticians (Singhal et al., 2023; Jiang et al., 2017).

Yet, as generative AI becomes more deeply embedded in the fabric of medical research (Singhal et al., 2023), it is essential to reflect on the longstanding challenges that have shaped the field, and chief among them is the misuse of statistics. The integrity of medical science depends on the correct application and interpretation of statistical methods. However, a substantial body of evidence has shown that statistical errors are both common and consequential in the biomedical literature.

A series of landmark investigations have illuminated the scope and persistence of statistical misapplication in biomedical research. In 1994, Douglas Altman's influential editorial in the BMJ exposed the widespread prevalence of basic statistical errors in published studies, even in leading journals, and underscored the urgent need for better statistical education and more rigorous peer review (Altman, 1994). A decade later, John Ioannidis's seminal paper in PLOS Medicine fundamentally challenged the credibility of the biomedical literature, demonstrating that a confluence of factors such as small sample sizes, selective reporting, flexible study designs, and, crucially, statistical misapplication renders most published research findings likely to be false (Ioannidis, 2005). More recent systematic reviews have confirmed that issues like p-hacking, selective reporting, and the misuse of *p*-values remain stubbornly prevalent in scientific literature (Nuijten et al., 2016; Chavalarias et al., 2016; Strasak et al., 2007).

As generative AI becomes an important tool in medical research, there is a genuine risk that these longstanding issues may not only persist but be amplified. The automation of statistical analysis and interpretation by AI models, if guided by poorly constructed prompts or used by individuals lacking statistical expertise, could lead to the rapid and widespread dissemination of erroneous results. AI-generated outputs, often presented with an air of authority and fluency, may make it even more difficult to detect subtle errors or misinterpretations. In this context, the responsible design and use of prompts, prompt engineering, emerges as a critical safeguard.

This paper critically examines the intersection of prompt engineering, generative AI, and medical statistics, with a focus on minimizing the risk of erroneous outputs that may exacerbate the longstanding issue of statistical misuse in biomedical research. By situating this discussion within the well-documented history of statistical errors, the paper underscores the importance of understanding and applying prompt engineering to guide AI models toward accurate and reliable results. Particular attention is given to the challenge of preventing AI-generated hallucinations and ensuring that outputs support, rather than undermine, sound statistical practice. To address these concerns, this study provides practical suggestions and general guidelines for avoiding erroneous responses when conducting basic statistical analyses and statistical inference using generative AI. In doing so, the paper aims to equip researchers with strategies that promote the responsible and effective integration of AI into medical research, safeguarding the integrity of scientific discovery. To the best of our knowledge, no prior study has systematically examined the role of prompt engineering in mitigating statistical errors within the context of generative AI applications in medical research. Given the increasing reliance on AI for statistical analysis, this oversight represents a critical gap in the literature. This paper seeks to bridge that gap by offering a structured evaluation of prompting strategies and their implications for statistical validity and interpretability.

## 2 Materials and methods

### 2.1 Prompt engineering

As generative AI systems become increasingly sophisticated, the art and science of “prompt engineering” has emerged as a central discipline for harnessing their capabilities. Prompt engineering refers to the deliberate design and formulation of input queries, prompts, to guide LLMs toward producing accurate, relevant, and contextually appropriate outputs (Liu et al., 2023). In essence, prompt engineering is the interface between human intent and machine intelligence, shaping how AI interprets and responds to complex tasks. A well-crafted prompt is more than a simple question; it is a structured communication that encodes the user's objectives, constraints, and expectations. The quality of a prompt can dramatically influence the reliability and utility of AI-generated responses, particularly in high-stakes domains such as medical statistics. As such, prompt engineering is not merely a technical exercise, but a critical component of responsible AI deployment (Brown et al., 2020; Reynolds and McDonell, 2021).

### 2.2 Guidelines and structure for effective prompts

The literature on prompt engineering has converged on several key guidelines for constructing effective prompts. Clarity is paramount: prompts should be unambiguous, specific, and free from unnecessary complexity. Contextual information such as the desired format of the output, relevant background, or explicit instructions should be included to minimize misinterpretation. Additionally, prompts should anticipate potential sources of confusion, such as ambiguous terminology or multiple possible interpretations, and address them directly (Zhou K. et al., 2022).

Structurally, effective prompts often include:

- A clear statement of the task or question.
- Any necessary context or background information.
- Explicit instructions regarding the format or style of the output.
- Stepwise guidance for multi-part or complex tasks.

These elements help ensure that the model's output aligns with the user's expectations and the requirements of the task at hand.

### 2.3 Approaches to prompt engineering

Prompt engineering encompasses a spectrum of strategies, each with distinct strengths and limitations. The choice of approach depends on the complexity of the task, the desired level of control, and the context in which the AI is deployed. Understanding these methods is essential for leveraging generative AI effectively in medical statistics.

#### 2.3.1 Zero-shot prompting

Zero-shot prompting is a foundational approach for interacting with LLMs, where the model is given a single, direct instruction without any examples. For instance, a prompt might read, “Summarize the results of this clinical trial.” In this setting, the model relies entirely on its pre-trained knowledge and generalization abilities to interpret and respond to the task (Kojima et al., 2022; Brown et al., 2020; Wei et al., 2022a).

The primary advantage of zero-shot prompting is its simplicity and efficiency. It requires minimal effort from the user and can be effective for well-defined, common tasks. However, its major limitation is unpredictability: without examples or additional context, the model may misinterpret the intent or produce outputs that lack the necessary specificity or rigor, especially in specialized domains like medical statistics (Brown et al., 2020; Wei et al., 2022a).

#### 2.3.2 Few-shot prompting

Few-shot prompting extends the zero-shot approach by supplying the model with a small number of examples that demonstrate the desired input-output relationship. For instance, a prompt might present two or three sample statistical analyses, each paired with its corresponding summary or interpretation, followed by a new case for the model to address in the same manner. By explicitly showing the model how to perform the task, few-shot prompting helps guide its responses toward the expected format and content (Brown et al., 2020; Wei et al., 2022a).

This method offers greater control over the model's output and can substantially enhance both the relevance and accuracy of responses, particularly for tasks that are less common or more complex. The inclusion of well-chosen examples allows the model to better understand the nuances of the task and adapt its output accordingly. However, the effectiveness of few-shot prompting depends heavily on the quality and representativeness of the examples provided. If the examples are ambiguous or not closely aligned with the intended task, the model's performance may suffer. Additionally, this approach may not scale efficiently to tasks with high variability, as it becomes challenging to provide examples that cover all possible scenarios (Brown et al., 2020; Wei et al., 2022a).

#### 2.3.3 Explicit, instruction-based prompting

Explicit, instruction-based prompting takes a more structured approach by providing the model with clear, detailed, and stepwise instructions. This method not only specifies the task but also outlines the sequence of actions and any necessary checks or conditions. For example, a prompt might state:

“First, check whether the data meet the assumptions for a *t*-test (normality and equal variances). If the assumptions are met, perform the *t*-test. If not, select and perform an appropriate alternative test. Report the results, including the test statistic, degrees of freedom, and *p*-value.”

By breaking down the workflow into explicit steps, this approach reduces ambiguity and guides the model through complex analytical processes. It helps ensure that critical steps such as verifying statistical assumptions are not overlooked, which is particularly important in medical and health research where methodological rigor is essential.

The primary advantage of explicit, instruction-based prompting is its ability to minimize errors of omission and misinterpretation. By clearly delineating each step, the model is less likely to skip important procedures or misapply statistical methods. However, this approach does require the user to have a solid understanding of the statistical process, as the quality and completeness of the instructions directly influence the reliability of the model's output (Ouyang et al., 2022; Naveed et al., 2023). This method is especially valuable in domains where precision and adherence to methodological standards are critical, supporting more reliable and transparent statistical analysis.

#### 2.3.4 Chain-of-thought prompting

Chain-of-thought (CoT) prompting is a technique that encourages the model to articulate its reasoning process step by step, making each intermediate stage of analysis explicit. For example, a prompt might instruct:

“Explain your reasoning step by step before giving the final answer.”

This approach is particularly effective for complex analytical tasks, as it reveals the model's logic and allows users to trace the sequence of decisions or calculations that lead to the final output (Wei et al., 2022b; Kojima et al., 2022). By surfacing the model's reasoning, CoT prompting can help identify errors or gaps in logic, supporting more robust and transparent analyses.

The primary advantage of chain-of-thought prompting is the increased transparency and reliability it brings to multi-step problems. Users can review each stage of the model's reasoning, making it easier to spot mistakes or misunderstandings. However, this method can result in verbose outputs and does not guarantee that every reasoning step is correct; the model may still make errors in logic or interpretation.

#### 2.3.5 Format-constrained prompting

Format-constrained prompting directs the model to present its output in a specific structure, such as a table, list, or code block. For instance, a prompt might state:

“Present the results in a table with headings: test statistic, df, *p*-value.”

This approach is especially valuable in medical statistics, where standardized reporting is essential for clarity and reproducibility. By specifying the desired format, format-constrained prompting minimizes the risk of unstructured or incomplete outputs and helps ensure that all relevant information is included. Nevertheless, the effectiveness of this method still depends on the model's underlying knowledge and its ability to follow formatting instructions (Ouyang et al., 2022; Naveed et al., 2023).

We summarize the different prompting approaches in Table 1.

**Table 1:** Comparison of prompt engineering approaches.

**Approach**	**Description**	**Advantages**	**Disadvantages**
Zero-shot prompting	Direct instruction, no examples	Simple, efficient	May lack specificity, unpredictable
Explicit, instruction-based	Clear, stepwise instructions	Reduces ambiguity, minimizes errors of omission	Requires user expertise, can be verbose
Few-shot prompting	Provides a few examples	Greater control, improved relevance	Sensitive to example quality, may not scale
Chain-of-thought prompting	Encourages stepwise reasoning	Transparent, better for complex tasks	Verbose, reasoning may not always be correct
Format-constrained prompting	Specifies output structure	Standardized, easy to interpret	Still relies on model's statistical knowledge

This table summarizes the main approaches to prompt engineering, their descriptions, advantages, and disadvantages.

### 2.4 Variants and hybrids in prompt engineering

As the field of prompt engineering has matured, practitioners have developed a range of variants and hybrid strategies that blend the strengths of multiple approaches (Giray, 2023). These innovations are particularly relevant in medical statistics, where both methodological rigor and clarity of communication are paramount (Wang and Zhang, 2024).

One important variant is zero-shot chain-of-thought prompting. Here, the user asks the model to “think step by step” even without providing examples. This simple instruction can significantly improve the model's reasoning, especially for complex statistical tasks. Another variant is zero-shot with context, where the prompt includes relevant background information or definitions to anchor the model's response more effectively (Kojima et al., 2022).

Few-shot prompting has also evolved. In contextual calibration, the examples provided are carefully selected to match the difficulty or style of the target task, ensuring the model's output is both relevant and accurate. Dynamic few-shot prompting adapts the set of examples in real time based on the model's previous outputs, allowing for a more responsive and tailored interaction (Zhang et al., 2025; Kojima et al., 2022).

Chain-of-thought prompting can be combined with few-shot learning to create few-shot chain-of-thought prompts. In this approach, the model is shown several examples of stepwise reasoning, which helps it internalize the logic and structure required for complex analyses. Format-constrained prompting can also be hybridized. For instance, a prompt might combine explicit instructions, a required output format (such as a table), and a few illustrative examples. This multi-layered approach maximizes clarity and minimizes the risk of misinterpretation (Wang and Zhou, 2024).

### 2.5 Limitations of prompt engineering

While the evolution of prompt engineering has introduced powerful variants and hybrid strategies, several limitations remain that constrain its broader applicability and reliability. These limitations are particularly salient in domains like medical statistics, where precision, reproducibility, and interpretability are essential (Lu et al., 2021).

One key limitation is the lack of generalizability. Prompts that perform well in one context may fail in another, especially when transferred across models or domains. This brittleness is compounded by the sensitivity of LLMs to minor changes in phrasing, formatting, or context (Bommasani et al., 2021).

Prompt engineering also relies heavily on trial-and-error. Designing effective prompts often requires iterative tuning, domain expertise, and manual curation of examples. This process can be time-consuming and may not scale efficiently for complex or evolving tasks (Liu et al., 2023).

Another challenge is the opacity of model behavior. Even with carefully crafted prompts, the internal reasoning of the model remains largely inaccessible, making it difficult to diagnose errors or ensure consistency. This lack of transparency can be problematic in high-stakes applications where interpretability is critical (Bommasani et al., 2021).

Moreover, prompt-based methods are susceptible to bias and hallucination. Without external grounding or verification, models may generate plausible-sounding but incorrect or biased outputs, especially when prompts are ambiguous or under-specified (Ouyang et al., 2022; Ji et al., 2023).

Finally, while hybrid strategies offer improved performance, they often require more complex prompt structures and greater user effort. This can introduce new sources of error and reduce usability, particularly for non-expert users (Wei et al., 2022b; Zhou D. et al., 2022).

### 2.6 Commonly used statistics in medical research

Descriptive and inferential statistics form the backbone of quantitative analysis in medical and health research. At the most fundamental level, descriptive statistics are employed to summarize and characterize data. Measures such as the mean, median, and mode are routinely used to describe central tendency, while standard deviation, interquartile range, and variance provide insight into data dispersion (Altman, 1990). However, the choice of summary statistic must be guided by the underlying distribution of the data. For instance, while the mean is a widely reported measure, it can be misleading in the presence of skewed distributions or outliers, where the median often provides a more robust summary (Hoaglin et al., 2000). Similarly, graphical representations such as histograms and boxplots are essential for visualizing data distributions and identifying anomalies.

Beyond description, inferential statistics enable researchers to draw conclusions about populations based on sample data. Commonly used inferential methods include hypothesis testing, confidence intervals, and regression analysis. The *t*-test and analysis of variance (ANOVA) are frequently applied to compare group means, while non-parametric alternatives such as the Mann-Whitney U test or Kruskal-Wallis test are appropriate when data do not meet the assumptions of normality or homogeneity of variance (Motulsky, 2014). Correlation and regression analyses are used to assess relationships between variables, with linear regression being a staple for modeling continuous outcomes and logistic regression for binary outcomes (Hosmer et al., 2013).

Despite their ubiquity, the application of these statistical methods is not without challenges. A common pitfall is the inappropriate use of parametric tests, such as the *t*-test, on data that violate key assumptions, leading to invalid inferences. The misuse of *p*-values, that is,interpreting statistical significance as evidence of clinical importance or failing to account for multiple comparisons remains a persistent issue in the literature (Wasserstein and Lazar, 2016). Additionally, selective reporting of significant results and p-hacking can distort the scientific record and undermine the credibility of research findings (Head et al., 2015). Table 2 summarizes some of the most frequent statistical pitfalls encountered in medical research.

**Table 2:** Common statistical pitfalls in medical research.

**Pitfall**	**Description**
Misuse of mean	Reporting the mean for skewed data instead of the median, which can misrepresent the true central tendency.
Inappropriate test selection	Using parametric tests (e.g., *t*-test, ANOVA) on data that do not meet assumptions of normality or equal variances, rather than opting for non-parametric alternatives.
*P*-value misinterpretation	Treating statistical significance as equivalent to clinical or practical significance, or misunderstanding what a *p*-value actually represents.
Multiple comparisons	Conducting multiple hypothesis tests without proper adjustment, which increases the risk of false-positive findings.
Selective reporting	Only presenting statistically significant results, leading to publication bias and an incomplete scientific record.

A careful and context-aware application of statistical methods is essential for producing valid and reproducible results in medical research. By recognizing the strengths and limitations of commonly used statistics, researchers can avoid common errors and contribute to the advancement of robust scientific knowledge.

### 2.7 Evaluating LLMs

The increasing integration of LLMs into biomedical and statistical workflows necessitates a rigorous and context-sensitive approach to evaluating their outputs. As these models are entrusted with tasks ranging from summarizing clinical findings to performing inferential statistical analyses, the question of how to assess their performance becomes not only technical but epistemological. Evaluation is no longer a peripheral concern; it is central to ensuring that LLMs function as reliable collaborators in scientific inquiry.

Historically, the evaluation of natural language generation (NLG) systems has relied on metrics that prioritize surface-level similarity to reference texts, such as BLEU and ROUGE. These metrics, while useful for tasks like translation or summarization, operate primarily on n-gram overlap and often fail to capture semantic correctness or contextual appropriateness (Faizullah et al., 2024). However, the emergence of LLMs which are capable of generating fluent, contextually rich, and often persuasive outputs has exposed the limitations of such metrics, particularly in tasks requiring reasoning or domain-specific accuracy (Faizullah et al., 2024). In high-stakes domains like medical statistics, where the correctness of an output cannot be inferred from its fluency alone, evaluation must account for methodological rigor, interpretive accuracy, and domain relevance. This section reviews the principal approaches to LLM evaluation, with a focus on their applicability to prompt engineering in statistical contexts.

#### 2.7.1 Manual evaluation

Manual evaluation involves human experts assessing the quality of LLM outputs based on predefined rubrics or subjective judgment. This approach is particularly valuable in domains requiring nuanced interpretation, such as statistical reasoning or clinical decision-making. Human evaluators can detect subtle errors, assess contextual appropriateness, and apply domain-specific standards that are difficult to encode algorithmically (Liu et al., 2023).

However, manual evaluation is inherently limited by its subjectivity, labor intensity, and lack of scalability. Inter-rater variability can compromise reliability, and the process is often too slow for iterative prompt development or real-time deployment (Liu et al., 2023). Despite these limitations, manual evaluation remains indispensable for validating automated methods and for assessing outputs in novel or ambiguous contexts where ground truth is unavailable or contested (Gao et al., 2025).

#### 2.7.2 Automated evaluation

Automated evaluation methods offer scalability and reproducibility, making them attractive for benchmarking and continuous integration. These methods fall into two broad categories: reference-based and reference-free.

Reference-based metrics such as BLEU, ROUGE, and METEOR compare model outputs to predefined reference texts, quantifying lexical overlap. While these metrics are useful for tasks like summarization or translation, they are poorly suited to evaluating statistical reasoning. Lexical similarity does not guarantee semantic correctness, and models may produce outputs that are superficially similar to reference texts while misrepresenting statistical assumptions or misapplying analytical methods (Lavie and Denkowski, 2009).

Reference-free methods address some of these limitations by evaluating outputs without relying on ground truth references. These include semantic similarity scoring using embedding models, rule-based validation of output structure, and the increasingly prominent approach of using LLMs themselves as evaluators; a method known as “LLM-as-a-judge” (Lavie and Denkowski, 2009).

#### 2.7.3 LLM-as-a-judge

The LLM-as-a-judge approach involves prompting a language model to assess the outputs of another (or the same) model based on a rubric or set of criteria. This method is particularly well-suited for evaluating prompt effectiveness, as it allows for rapid, rubric-based comparison of different prompt formulations (Berti et al., 2024). For example, an evaluator model can be instructed to assess outputs based on assumption checking, test selection, output completeness, and interpretive quality–criteria that are central to statistical validity.

This approach offers several advantages. It is highly scalable, enabling the evaluation of thousands of prompt-output pairs without human intervention. It also allows for nuanced assessments that align more closely with human judgment, particularly when the evaluator model is guided by explicit instructions or chain-of-thought reasoning (Shankar et al., 2024). Moreover, it facilitates iterative prompt refinement by providing structured feedback on the strengths and weaknesses of different prompt designs.

However, the method is not without limitations. A primary concern is the potential for bias and circularity, especially when the evaluator shares architecture or training data with the model being evaluated. This can lead to inflated performance estimates or the reinforcement of shared misconceptions (Berti et al., 2024). Additionally, the internal reasoning of the evaluator model is often opaque, making it difficult to audit or interpret its judgments, an issue that is particularly problematic in clinical and statistical domains where transparency is essential (Shankar et al., 2024).

Another challenge lies in domain specificity. While general-purpose LLMs may perform well in evaluating outputs related to everyday language tasks, their ability to assess domain-specific outputs such as the appropriateness of a statistical test or the validity of an inferential conclusion depends on their exposure to relevant training data and their capacity for domain-specific reasoning. In such cases, hybrid evaluation pipelines that combine LLM-based and human assessments may offer a more robust solution (Berti et al., 2024).

Recent empirical studies support the utility of LLM-as-a-judge in scientific contexts. (Gao et al. 2025) demonstrated that LLM-based evaluators can reliably distinguish between high- and low-quality outputs in natural language generation tasks, particularly when guided by structured rubrics. Similarly, (Kojima et al. 2022) and (Wei et al. 2022b) have shown that chain-of-thought prompting enhances the reasoning capabilities of LLMs, both as generators and as evaluators.

#### 2.7.4 Hybrid evaluation: combining LLM-as-a-judge with human oversight

As LLMs become increasingly embedded in scientific and statistical workflows, the need for evaluation frameworks that balance scalability with interpretive rigor has become more urgent. While automated methods such as LLM-as-a-judge offer efficiency and consistency, they are not immune to issues of bias, opacity, and domain mismatch. Conversely, manual evaluation provides depth and contextual sensitivity but lacks scalability and reproducibility. To address these limitations, recent research has proposed hybrid evaluation frameworks that integrate both approaches in a complementary manner (Shahzad et al., 2025).

In a hybrid framework, the evaluation process typically unfolds in two stages. First, an LLM is prompted to assess the outputs of another model or the same model under different prompt conditions using a structured rubric. This rubric may include criteria such as assumption checking, test selection, output completeness, and interpretive quality. The LLM's evaluations are recorded and scored, providing a scalable and rubric-aligned first-pass assessment of prompt effectiveness (Kamath et al., 2024).

In the second stage, human experts review the LLM's evaluations. This part serves to validate the model's judgments, identify potential hallucinations or misinterpretations, and refine the evaluation rubric based on observed model behavior. This human-in-the-loop process is particularly valuable in domains like medical statistics, where subtle errors in reasoning or misapplication of statistical methods can have significant consequences. The hybrid approach thus combines the breadth of automated evaluation with the depth of expert review, enabling both high-throughput assessment and epistemic accountability (Kumar, 2024).

This framework has been shown to improve the reliability and transparency of LLM evaluations, particularly when applied to tasks involving complex reasoning or domain-specific knowledge. It also supports iterative prompt refinement, as human reviewers can use the LLM's feedback to identify patterns of failure or success across different prompt formulations. Moreover, by documenting both the LLM's evaluations and the human corrections, the framework promotes reproducibility and provides a valuable audit trail for future research and model development (Shahzad et al., 2025). A comparison of the LLM evaluation approaches is given in Table 3.

**Table 3:** Comparison of LLM evaluation approaches.

**Evaluation approach**	**Description**	**Advantages**	**Disadvantages**
Manual evaluation	Human experts assess the quality of LLM outputs based on predefined rubrics or subjective judgment.	Provides depth and contextual sensitivity; can detect subtle errors and apply domain-specific standards.	Subjective, labor-intensive, and lacks scalability; inter-rater variability can compromise reliability.
Reference-based evaluation	Metrics such as BLEU, ROUGE, and METEOR compare model outputs to predefined reference texts, quantifying lexical overlap.	Objective and reproducible; suitable for benchmarking and regression testing.	Poorly suited to evaluating statistical reasoning; lexical similarity does not guarantee semantic correctness.
Reference-free evaluation	Evaluates outputs without relying on ground truth references, using techniques like semantic similarity scoring, rule-based checks, or LLMs as evaluators.	Flexible and adaptable to real-world use cases; enables continuous monitoring and quality control.	Challenges related to reliability, interpretability, and standardization; embedding-based measures can be opaque.
LLM-as-a-judge	An LLM is prompted to assess the outputs of another (or the same) model based on a rubric or set of criteria.	Highly scalable; allows for nuanced, rubric-based assessments; facilitates iterative prompt refinement.	Potential for bias and circularity; internal reasoning is often opaque; effectiveness varies across domains.
Hybrid evaluation	Combines LLM-as-a-Judge with human oversight, where LLMs provide initial evaluations and human experts review and refine these assessments.	Balances scalability with interpretive rigor; supports high-throughput assessment and epistemic accountability.	Requires more complex prompt structures and greater user effort; potential for new sources of error.

This table summarizes the main approaches to evaluating large language models, including their descriptions, advantages, and disadvantages.

In this paper, we adopt a hybrid evaluation framework to assess the effectiveness of various prompt engineering strategies for statistical analysis. Specifically, outputs generated under different prompting conditions were evaluated by Microsoft Copilot and subsequently reviewed by the author. The human review involved cross-validation using standard statistical software and focused on four key criteria: assumption checking, test selection, output completeness, and interpretive quality. While a single expert reviewer was used for consistency in this study, future research should incorporate multiple reviewers and consensus-based evaluation methods to enhance reproducibility and reduce bias, as recommended in recent literature on LLM evaluation frameworks (Gao et al., 2025; Shahzad et al., 2025).

## 3 Results

If not prompted correctly, generative AI systems can produce misleading or incomplete responses. The quality and reliability of outputs from LLMs are highly dependent on the clarity and specificity of the input prompts. As such, effective prompt design is essential for obtaining accurate and contextually appropriate results.

With a growing number of generative AI models available, it is the user's responsibility to select the most suitable model for the task at hand. Models such as OpenAI's GPT-4, Anthropic's Claude, and Microsoft Copilot have demonstrated strong capabilities in statistical reasoning, as well as in generating code for languages like R and Python. These models can be powerful tools for data analysis, but their effectiveness hinges on how well they are guided through prompting.

### 3.1 Descriptive statistics

Descriptive statistics are typically the first step in data analysis, especially for numerical datasets. Measures of central tendency–such as the mean and median–and measures of dispersion–such as the standard deviation and interquartile range (IQR) are commonly used to summarize data characteristics.

For these tasks, we recommend the use of zero-shot prompting. A well-structured instruction specifying the required statistical measures is often sufficient to elicit accurate and complete responses from LLMs. This approach assumes that the user has a basic understanding of statistical concepts and knows what they intend to compute.

We also advise users to assess the distribution of the data particularly its normality before interpreting descriptive statistics. This can be done using a simple zero-shot prompt.

To illustrate, consider the following artificial dataset:


  56.31, 361.21, 158.01, 109.55, 20.35, 20.35,
  7.18, 241.35, 110.29, 147.75


This dataset is clearly right-skewed. There are multiple ways to prompt an LLM to analyze such data. One effective zero-shot prompt is:

Calculate the mean, median, standard deviation and the interquartile range for the data: 56.31, 361.21, 158.01, 109.55, 20.35, 20.35, 7.18, 241.35, 110.29, 147.75. Is the data skewed?

This prompt is explicit and comprehensive. It instructs the model to compute all relevant statistics and includes a final question to guide interpretation specifically whether the mean or median is more appropriate to report.

Alternatively, a more abstract yet effective prompt is:

Describe the data using measures of central tendency and dispersion. Looking at the distribution of the data, which measures should I report?

We prefer this formulation. It is specific in its request for descriptive statistics but does not require the user to name individual metrics. This makes it accessible to users with limited statistical background. Moreover, it leverages the model's reasoning capabilities to recommend appropriate measures based on the data distribution. This prompt was tested across multiple LLMs, including Claude 3.7 Sonnet, GPT-4.1, and Microsoft Copilot, and consistently produced accurate and context-aware responses.

Other descriptive measures such as range, variance, skewness, and kurtosis can also be computed using similar zero-shot prompts. We recommend the use of zero-shot prompting for generating descriptive statistics, as it is both efficient and reliable when the instructions are clearly formulated. Providing explicit and unambiguous instructions minimizes the risk of erroneous outputs and ensures that the statistics generated are appropriate for the intended context, whether for academic research, reporting, or exploratory data analysis.

The overarching goal is to avoid misleading interpretations and to ensure that the statistical summaries produced by the language model are both accurate and contextually relevant.

### 3.2 Inferential statistics

Caution is warranted when using generative AI for statistical inference in medical research. While LLMs can assist with data analysis, they are prone to producing erroneous outputs, particularly in inferential contexts. A key limitation lies in their handling of statistical assumptions, a foundational aspect of most inferential procedures.

Nearly all statistical models used for inference rely on specific assumptions (e.g., normality, homogeneity of variance, independence). When these assumptions are violated, alternative methods such as data transformation or non-parametric tests should be employed. For instance, the independent samples Student's *t*-test assumes normality and equal variances; if these are not met, a non-parametric alternative like the Mann-Whitney U test is more appropriate. Generative AI models may overlook or misinterpret these assumptions unless explicitly guided.

#### 3.2.1 Prompt evaluation for statistical inference

To assess the effectiveness of the various prompting strategies, we employed a hybrid evaluation framework. Two illustrative case studies are presented. In the first scenario, the assumption of equal variances is violated, necessitating the use of a *t*-test that accommodates unequal variances. In the second example, both the assumptions of normality and homogeneity of variances are breached, thereby warranting the application of a non-parametric alternative to the *t*-test. Table 4 outlines the specific prompts utilized in the first case.

**Table 4:** Prompts used for illustration I.

**Prompt type**	**Prompt**
Zero-shot	Perform a *t*-test on the following blood pressure data from a clinical trial [*data*].
Explicit instruction	I want to compare blood pressure between two groups [*data*]. Step 1: test for normality in each group. Step 2: test for equality of variances. Step 3: based on the results, choose the appropriate *t*-test. Step 4: perform the test and report the test statistic, degrees of freedom, and *p*-value. Step 5: interpret the result in plain language.
CoT	Consider the following blood pressure data [*data*]. Think step by step: • Are the data normally distributed? • Are variances equal? • Which test should be used? • What are the results? • What do they mean?
Hybrid	Analyze the following blood pressure data from a clinical trial [*data*]. Please: • Test for normality and equal variances. • Choose the correct test (Student's or Welch's *t*-test). • Report results in a table with columns: test statistic, df, *p*-value. • Provide a plain-language interpretation of the findings.

This table summarizes the approaches and the actual prompts used in analyzing the artificial blood pressure data. [*data*] indicates where the data should be placed.

#### 3.2.2 Illustrations

##### 3.2.2.1 Illustration I

Artificial data are generated for a blood pressure clinical trial where 40 patients were assigned between a new drug and a control treatment in the ratio 1:1. The data generation process is as follows:


  control_bp  < - rnorm(n_control, mean=150, sd=10)
  treatment_bp  < - rnorm(n_treat, mean=140, sd=20)


and the generated data is:


  control_bp = c(142.9, 152.6, 147.5, 146.5,
               140.5, 149.5, 142.2, 133.3,
               146.2, 159.2, 144.2, 156.1,133.8,
               149.4, 155.2, 153.0, 151.1,
               143.6, 141.5, 139.8)
  treatment_bp = c(122.4, 101.1, 110.2, 114.9,
               156.9, 107.0, 124.7, 121.6,
               100.8, 118.6, 148.9, 129.0,
               120.8, 111.6,  78.9, 142.6,
               90.8, 134.8, 158.2, 91.1)


##### 3.2.2.2 Illustration II

We further demonstrate the impact of prompt engineering using some artificial BMI data from a 12-week randomized controlled trial comparing a plant-based diet intervention to standard care among primary school children with elevated BMI. The data are as follows:


 
  control_bmi = c(22.3, 23.3, 31.2, 24.2, 24.4,
  29.1, 33.8, 20.2, 21.9, 22.7, 27.7, 25.1,
  25.2, 24.3, 52.3)
  treatment_bmi = c(23.1, 21.3, 24.2, 24.2, 24.1,
  23.7, 23.4, 21.8, 21.2, 21, 20.3, 21.5, 18.8,
  27.4, 25)
 


Table 5 shows the different prompts used in the second case study.

**Table 5:** Prompts used for illustration II.

**Prompt type**	**Prompt**
Zero-shot	Analyze the following BMI data from a randomized controlled trial [*data*].
Explicit instruction	I want to compare BMI between two groups [*data*]. Step 1: Test for normality in each group. Step 2: Test for equality of variances. Step 3: If assumptions are violated, use a non-parametric test . Step 4: Report the test statistic and *p*-value. Step 5: Interpret the result in plain language.
CoT	Consider the following BMI data [*data*]. Think step by step: • Are the data normally distributed? • Are variances equal? • Which test should be used? • What are the results? • What do they mean?
Hybrid	Analyze the following BMI data from a 12-week dietary intervention trial [*data*]. Please: • Test for normality and equal variances. • Can a *t*-test be used? • Perform an appropriate test. • Present results in a table with: test name, test statistic, *p*-value. • Provide a plain-language interpretation.

This table summarizes the approaches and the actual prompts used in analyzing the artificial BMI data. The data was left out from the prompts. [*data*] indicates where the data should be placed.

The evaluation results presented in Table 6 provide a comparative analysis of how different prompting strategies influence the performance of LLMs in conducting inferential statistical analyses. These tables summarize the outputs of two LLMs; GPT-4.1, and Claude 3.7 Sonnet. Microsoft Copilot was used as a judge to evaluate the outputs from GPT-4.1, and Claude 3.7 Sonnet. The two AI models were tasked with analyzing two artificial datasets using four distinct prompting strategies: zero-shot, explicit instruction-based, chain-of-thought, and hybrid prompting. The evaluation was conducted using a structured rubric that assessed four key dimensions of statistical reasoning: assumption checking, test selection accuracy, output completeness, and interpretive quality. The rubric is shown in Table A1.

**Table 6:** Evaluation summary by prompt type and model for illustration I and illustration II.

**Criterion**	**Zero-shot**		**Explicit instruction**		**Chain-of-thought**		**Hybrid**	
	**GPT-4.1**	**Claude 3.7 Sonnet**	**GPT-4.1**	**Claude 3.7 Sonnet**	**GPT-4.1**	**Claude 3.7 Sonnet**	**GPT-4.1**	**Claude 3.7 Sonnet**
**Illustration I**								
Assumption checking	0.0	0.0	1.0	1.0	1.0	1.0	1.0	1.0
Test selection	0.6	0.6	1.0	1.0	1.0	1.0	1.0	1.0
Output completeness	1.0	1.0	1.0	1.0	0.8	0.8	0.8	0.8
Interpretive quality	0.8	1.0	1.0	1.0	1.0	1.0	1.0	1.0
Total ccore	2.4	2.6	4.0	4.0	3.8	3.8	3.8	3.8
**Illustration II**								
Assumption checking	0.0	1.0	1.0	1.0	1.0	1.0	1.0	1.0
Test selection	0.0	1.0	1.0	1.0	1.0	1.0	1.0	1.0
Output completeness	0.6	1.0	1.0	1.0	1.0	1.0	1.0	1.0
Interpretive quality	0.6	1.0	1.0	1.0	1.0	1.0	1.0	1.0
Total score	1.2	4.0	4.0	4.0	4.0	4.0	4.0	4.0

In both case studies, the zero-shot prompting strategy consistently yielded the lowest scores across all models. For the blood pressure dataset (second part of Table 6), zero-shot prompts failed to elicit any assumption checking from the models, resulting in a score of 0.0 for that criterion. Test selection was partially correct in some instances, with scores ranging from 0.6 to 1.0, but the absence of diagnostic checks undermined the methodological soundness of the analyses. Output completeness and interpretive quality were somewhat better, with scores ranging from 0.8 to 1.0, indicating that while the models could produce fluent and structured outputs, these outputs were not always grounded in appropriate statistical reasoning.

In contrast, the explicit instruction-based, chain-of-thought, and hybrid prompting strategies consistently achieved higher scores across all criteria. These strategies prompted the models to perform assumption checks, select appropriate statistical tests based on those checks, and provide comprehensive and contextually relevant interpretations. For instance, in the BMI dataset (Table 6), all three models correctly identified the non-normal distribution in the control group and the unequal variances between groups when guided by explicit or hybrid prompts. Consequently, they selected the Mann-Whitney U test, a non-parametric alternative appropriate for the data structure. These prompts resulted in perfect scores (4.0) across all evaluation dimensions, demonstrating the effectiveness of structured and context-rich prompting in eliciting statistically valid outputs from LLMs.

Not shown here are the results obtained using Claude 4 Sonnet and Claude 4 Opus, which were consistent with those of Claude 3.7 Sonnet across all prompting strategies and evaluation criteria. Similarly, OpenAI's o3 mini and o4 mini models produced outputs that matched those of GPT-4.1 in both descriptive and inferential tasks. These findings suggest that the observed improvements in statistical reasoning are not limited to specific model versions but are generalizable across newer iterations of both Anthropic's Claude and OpenAI's GPT families. This consistency reinforces the central conclusion that prompt design, rather than model architecture alone, is the primary determinant of output quality in statistical contexts.

Table 7 provides the summary of assumption checks and statistical test results for BMI data.

**Table 7:** Summary of assumption ahecks and statistical test results for BMI data.

**Test**	**Data**	**Statistic**	***p*-value**	**Interpretation**
Shapiro-Wilk (Control)	Original	*W* = 0.701	0.00025	Not normal
Shapiro-Wilk (Treatment)	Original	*W* = 0.970	0.853	Normal
F-test for variances	Original	*F* = 13.127	2.09 × 10^−5^	Variances unequal
Mann-Whitney U	Original	*W* = 171	0.0161	Significant

#### 3.2.3 A prompting strategy for inferential problems

We propose a structured prompting strategy to guide the use of generative AI in statistical inference, particularly in sensitive domains such as medical research. The goal is to minimize errors and ensure that statistical procedures are applied appropriately. The following steps outline a recommended workflow:

- Upload the dataset to the chosen AI assistant or LLM interface, provide context.
- Select the appropriate statistical test for the research question. If uncertain, the user may prompt the AI for guidance on test selection.
- Test the assumptions underlying the selected statistical procedure (e.g., normality, homogeneity of variance, independence).
- If assumptions are met, proceed with the selected test.
- If assumptions are violated, prompt the AI to suggest and evaluate alternative procedures (e.g., non-parametric tests).
- If the alternative test's assumptions are met, perform the alternative test.
- Specify the desired output format, such as summary tables, *p*-values, confidence intervals, or effect sizes.
- Request an interpretation of the results in plain language, suitable for the intended audience.
- Validate the results by cross-checking with statistical software or consulting a domain expert.

The above workflow is summarized in the Table 8. The process begins with a clear definition of the research question or analytical objective. This foundational step ensures that all subsequent actions are aligned with the intended goal. Once the objective is established, the next step is to select a candidate statistical model or analytical approach that is appropriate for the data and the research question at hand.

**Table 8:** Summary of the prompt engineering steps for inferential problems.

**Step**	**Description**	**Decision/next action**
1	Define research question or analytical objective and upload data into AI assistant	—
2	Select candidate statistical model or analytical approach	—
3	Test assumptions of the selected model	Are assumptions satisfied?
4	If yes: formulate precise, context-rich prompt for the AI	Proceed to prompt construction
5	If no: search for alternative model and repeat assumption testing	Iterate until suitable model is found
6	Submit prompt to generative AI system	—
7	Evaluate AI output for accuracy, completeness, and format adherence	Is output satisfactory?
	If yes: incorporate output into research workflow	—
9	If no: revise prompt and resubmit	Repeat as necessary

After selecting a model, it is essential to rigorously test its underlying assumptions. This step acts as a critical checkpoint: if the assumptions are satisfied, the process moves forward to constructing a precise and context-rich prompt for the AI. This prompt should clearly specify the statistical method, the expected output format, and any relevant contextual details to guide the AI's response.

If, however, the model's assumptions are not met, the process does not proceed. Instead, an alternative model is sought, and its assumptions are tested in turn. This iterative process continues until a suitable model is identified.

Once a suitable model is confirmed and the prompt is constructed, the prompt is submitted to the generative AI system. The output generated by the AI is then carefully evaluated for accuracy, completeness, and adherence to the specified format. If the output meets the required standards, it is incorporated into the research workflow. If not, the prompt may be revised for greater clarity or specificity, and the process is repeated as necessary.

This structured approach ensures methodological rigor and effective communication with generative AI systems, supporting robust and transparent scientific analysis.

## 4 Discussion

The results of the two illustrative case studies underscore a critical insight: zero-shot prompting is insufficient for conducting inferential statistical analyses using generative AI. While zero-shot prompts may be adequate for basic descriptive tasks, as demonstrated in earlier sections of this paper, they fall short when applied to more complex analytical workflows that require methodological rigor. The absence of assumption checking in almost all zero-shot outputs is particularly concerning, as it reflects a fundamental gap in the models' ability to autonomously initiate diagnostic procedures without explicit instruction. This limitation aligns with findings from (Kojima et al. 2022), who observed that LLMs often default to surface-level heuristics in the absence of structured guidance, leading to shallow or incorrect reasoning in complex tasks.

The superior performance of explicit instruction-based, chain-of-thought, and hybrid prompting strategies highlights the importance of prompt specificity and structure in guiding LLMs toward valid statistical reasoning. These strategies not only improved the accuracy of test selection but also enhanced the completeness and interpretive quality of the outputs. The inclusion of stepwise instructions and reasoning cues enabled the models to navigate the analytical process more transparently, mirroring the benefits of chain-of-thought prompting reported by (Wei et al. 2022b), who demonstrated that such prompts significantly enhance the reasoning capabilities of LLMs across a range of tasks. Moreover, the hybrid prompting strategy, which combines elements of explicit instruction, reasoning scaffolds, and format constraints, emerged as particularly effective. This approach ensured that the models adhered to methodological standards while also producing outputs that were interpretable and aligned with clinical relevance. The robustness of this strategy across both case studies suggests that hybrid prompting may serve as a best-practice framework for integrating LLMs into medical statistical workflows. This finding is consistent with recent work by (Wang and Zhang 2024), who advocate for multi-layered prompting strategies to maximize the reliability and interpretability of AI-generated outputs in healthcare contexts (Wang et al., 2022).

Few-shot prompting was excluded from the evaluation due to its reliance on curated examples, which introduces variability and complicates standardization. Although it has demonstrated effectiveness in other domains, its utility in medical statistics is highly dependent on the quality and relevance of the examples provided. These constraints make it less suitable for the structured and reproducible evaluation framework adopted in this study.

The consistency of results across different LLMs further reinforces the generalizability of these findings. Although some models, such as Claude 3.7 Sonnet, demonstrated slightly more comprehensive outputs under minimal prompting, all models benefited significantly from structured prompts. This suggests that the observed improvements are not model-specific but rather a function of prompt design, a conclusion that echoes the broader literature on prompt engineering as a critical interface between human intent and machine intelligence (Liu et al., 2023).

The evaluation results provide compelling evidence that prompt engineering is not merely a technical convenience but a methodological necessity in the application of generative AI to medical statistics. The ability of LLMs to produce valid, complete, and contextually appropriate analyses is highly dependent on the clarity, specificity, and structure of the prompts they receive. As such, researchers and practitioners must approach prompt design with the same rigor as they would any other component of the analytical process, particularly in high-stakes domains where the consequences of statistical error are profound.

In this study, the outputs generated by GPT-4.1 and Claude 3.7 Sonnet were evaluated using Microsoft Copilot as the LLM-as-a-judge. These evaluations were subsequently reviewed by the author. Notably, there was complete agreement between the human assessments and those generated by Copilot across all evaluation criteria. This alignment reinforces the reliability of the LLM-as-a-judge approach in this context and supports its use as a scalable yet accurate evaluation method for prompt engineering in statistical tasks. The observed concordance may be attributed to the inherently objective nature of statistical reasoning, where tasks such as assumption checking, test selection, and interpretation follow well-defined rules and diagnostic criteria. In such domains, where procedural clarity is high and ambiguity is minimal, LLMs are more likely to replicate expert-level judgment. This suggests that LLM-as-a-judge frameworks may be particularly effective in evaluating outputs in structured analytical domains like medical statistics, where correctness can be assessed against established methodological standards.

While this study demonstrates the potential of prompt engineering to improve statistical reasoning in LLMs, several limitations warrant attention. First, the reliance on artificial datasets, while methodologically convenient, may not fully reflect the complexity and variability of real-world medical data. Second, prompt brittleness remains a challenge, minor changes in phrasing can lead to divergent outputs, undermining reproducibility. Third, LLMs are prone to hallucinations, generating plausible but incorrect results, especially when prompts are underspecified or ambiguous. These risks are amplified in medical contexts, where statistical misinterpretation can have serious implications for clinical decision-making and public health.

Importantly, there is a growing concern about blind trust in AI-generated outputs. The fluency and confidence with which LLMs present information may obscure underlying errors, leading users to accept results without adequate scrutiny. As highlighted by (Ji et al. 2023) and (Shankar et al. 2024) embedding human oversight and promoting transparency are essential safeguards. Researchers must treat LLMs as assistive tools, not authoritative sources, and validate outputs using domain expertise and statistical software. Future work should explore ethical frameworks and governance models for responsible AI deployment in medical research, ensuring that technological innovation does not compromise scientific integrity.

## 5 Conclusion

This paper has situated the integration of generative AI within the broader context of medical statistics, emphasizing the dual potential of LLMs to either enhance or undermine statistical rigor depending on the quality of prompt design. As the biomedical research community increasingly turns to LLMs for assistance with data analysis, the findings presented here underscore the critical importance of prompt engineering as a methodological safeguard against statistical misinterpretation.

Among the prompting strategies evaluated, hybrid prompting; defined as the combination of explicit instruction, chain of thought reasoning, and format constraints proved to be the most effective approach for inferential statistical tasks. This strategy consistently guided LLMs to perform assumption checks, select appropriate tests, and produce outputs that were both methodologically sound and interpretively coherent. In contrast, zero shot prompting, while adequate for basic descriptive statistics, was insufficient for tasks requiring analytical nuance and diagnostic rigor.

The evaluation also revealed that while different LLMs such as GPT 4.1, and Claude 3.7 Sonnet varied slightly in their baseline performance, all models demonstrated substantial improvements when guided by structured prompts. This suggests that the quality of prompt design, rather than model architecture alone, is the primary determinant of output reliability in statistical contexts.

Building on the findings of this study, future research should explore the application of prompt engineering strategies in more complex statistical domains such as survival analysis, regression modeling, longitudinal data analysis, and meta-analysis. These areas present additional challenges in assumption checking, model selection, and interpretation, and would provide a more rigorous test of LLM capabilities. A dedicated investigation into these advanced methods would help determine whether the benefits of structured prompting generalize beyond basic inferential procedures.

Additionally, future work should incorporate real-world clinical or epidemiological datasets to assess how LLMs handle the variability, noise, and missingness typical of medical data. The integration of few-shot prompting strategies particularly when high-quality exemplars are available also warrants investigation, despite the practical challenges of prompt length and variability. To enhance reproducibility and reduce bias, future studies should involve multiple expert reviewers and consensus-based evaluation methods. Finally, the development of standardized prompt templates, automated prompt-generation tools, and domain-specific evaluation rubrics will be essential to support reproducibility, usability, and ethical deployment of LLMs in medical research. These efforts should be accompanied by robust human oversight frameworks to mitigate risks associated with hallucinations, prompt brittleness, and over-reliance on AI-generated outputs.

## Data Availability

The original contributions presented in the study are included in the article/supplementary material, further inquiries can be directed to the corresponding author.

## Appendix

**Table A1:** LLM evaluation scoring rubric.

**Criterion**	**Score**	**Description**
Assumption checking	1.0	Correctly identifies and tests all relevant assumptions (e.g., normality, variance)
Assumption checking	0.8	Tests most assumptions but misses one minor check
Assumption checking	0.6	Tests some assumptions but overlooks key ones
Assumption checking	0.4	Mentions assumptions but does not test them
Assumption checking	0.2	Incorrectly assumes assumptions are met or ignores them entirely
Assumption checking	0.0	No mention or testing of assumptions
Test selection accuracy	1.0	Selects the correct statistical test based on data type and assumptions
Test selection accuracy	0.8	Selects a mostly appropriate test with minor justification issues
Test selection accuracy	0.6	Selects a plausible but suboptimal test
Test selection accuracy	0.4	Selects an incorrect test but explains reasoning
Test selection accuracy	0.2	Selects an incorrect test with no justification
Test selection accuracy	0.0	No test selected or completely irrelevant test
Output completeness	1.0	Provides all key outputs (e.g., test statistic, p-value, CI, group means)
Output completeness	0.8	Provides most outputs but omits one or two minor elements
Output completeness	0.6	Provides partial output with some missing key elements
Output completeness	0.4	Output is vague or lacks structure
Output completeness	0.2	Output is incomplete and unclear
Output completeness	0.0	No usable output provided
Interpretive quality	1.0	Interpretation is accurate, context-aware, and clinically relevant
Interpretive quality	0.8	Interpretation is mostly accurate with minor contextual gaps
Interpretive quality	0.6	Interpretation is generic or lacks depth
Interpretive quality	0.4	Interpretation is partially incorrect or misleading
Interpretive quality	0.2	Interpretation is mostly incorrect or irrelevant
Interpretive quality	0.0	No interpretation provided
